# Age-related differences in conversational discourse abilities A comparative study

**DOI:** 10.1590/1980-57642018dn13-010006

**Published:** 2019

**Authors:** Natalie Pereira, Ana Paula Bresolin Gonçalves, Mariana Goulart, Marina Amarante Tarrasconi, Renata Kochhann, Rochele Paz Fonseca

**Affiliations:** 1 Doctoral student, Pontifícia Universidade Católica do Rio Grande do Sul, Porto Alegre, RS, Brazil.; 2 Psychology undergraduate student, Pontifícia Universidade Católica do Rio Grande do Sul, Porto Alegre, RS, Brazil.; 3 PhD, Pontifícia Universidade Católica do Rio Grande do Sul, Porto Alegre, RS, Brazil.

**Keywords:** conversational discourse, aging, neuropsychological assessment, communication, discurso conversacional, envelhecimento, avaliação neuropsicológica, comunicação

## Abstract

Conversational discourse (CD) is among the most complex tasks in everyday life and relies on multiple cognitive domains (communicative and executive abilities). Alterations in discourse comprehension and production are often present in pathological aging. However, there is still a need to identify changes in healthy aging.

**Objective:**

This study aimed to compare young and older adults for the frequency of impaired communicative behaviors on a CD task. Performance was scored according to the Complementary Procedure of Conversational Discourse Analysis (CPCDA), developed based on the CD task from the Montreal Communication Evaluation Battery.

**Methods:**

A total of 95 participants (54 young-adults and 41 older adults) were evaluated. The frequency of communicative behaviors was compared between groups using MANCOVA and Chi-square tests.

**Results:**

Young adults showed fewer impairments in expression, pragmatics, cohesion, coherence, comprehension and emotional prosody. Older adults showed higher levels of verbal initiative and had fewer word finding difficulties. Communicative behaviors associated with planning and self-monitoring (e.g. repetition of information and syllabic false starts) appear to be common in the speech of healthy individuals in general.

**Conclusion:**

Studies which evaluate both discursive and cognitive skills are required to identify age-related changes. This would allow for the development of screening tools for CD assessment and preventive programs.

## RESUMO.

O discurso conversacional (DC) está entre as tarefas diárias mais complexas e dependentes de múltiplos domínios cognitivos (habilidades comunicativas e executivas). Alterações na compreensão e produção do discurso são relatadas classicamente durante o envelhecimento patológico. No entanto, ainda é necessário esclarecer mudanças no envelhecimento saudável.

**Objetivo:**

Este estudo tem como objetivo comparar jovens adultos e idosos quanto à frequência de comportamento comunicativo desviante em uma tarefa de DC utilizando o Procedimento Complementar de Análise do Discurso Conversacional (PCADC), inspirado na tarefa de DC da Bateria Montreal de Avaliação da Comunicação.

**Métodos:**

Um total de 95 indivíduos (54 adultos jovens e 41 idosos) foram avaliados. A frequência dos comportamentos comunicativos desviantes foi comparadas entre os grupos usando análise MANCOVA e Qui-quadrado.

**Resultados:**

Adultos jovens apresentaram melhor desempenho nas habilidades comunicativas referentes à: expressão, pragmática, coesão, coerência, compreensão e linguística prosódica e emocional. O grupo de idosos obteve melhor desempenho nas variáveis: “falta de iniciativa verbal” e “procura ou troca palavra” do que os jovens. Itens associados ao planejamento da fala e auto-monitoramento (ex: “repete informações” e “realiza false start”), parecem estar associados a um comportamento comum na fala de indivíduos saudáveis em geral.

**Conclusão:**

Estudos que avaliem habilidades discursivas e cognitivas são necessários para identificar mudanças ​​influenciadas pela idade. Dessa forma, seria possível propor uma ferramenta de triagem para avaliação discursiva, bem como programas de intervenção preventiva.

The ability to hold a conversation with others is essential for participation in society, especially when elderly individuals are concerned.^1^ However, the fact that some elderly adults present changes in discourse comprehension and production is well-established in the literature.^2^ Nevertheless, these and other abilities related to social interaction are crucial for maintaining quality of life in aging.^3^ Though discourse tasks have been extensively used to investigate communication impairments across the life span,^4^^-^ 7 they are still underused by speech therapists and neuropsychologists in clinical settings.^8^ This occurs despite the fact that conversational discourse (CD) is among the most complex cognitive skills that humans can learn,^9^^,^ 10 as it involves a variety of cognitive process, including attention, executive functions^11^ and episodic memory.^12^

Discourse tasks can be used to evaluate several aspects of speech (such as pragmatic, syntactic, and grammatical features) in narrative or procedural discourse, as well as story recounting, picture descriptions and discursive speech.^4^ Narrative and procedural discourse are the most commonly studied, and often compared between healthy younger and older adults.^3^^,^ 4 ^,^ 13 ^,^ 14 Individual differences in discourse skills may depend on the type of discourse investigated.^4^^,^ 6 CD seems to be the least extensively studied, possibly due to the complexity of its analysis. However, some studies have already shown its relevance in conditions such as traumatic brain injury,^15^ Alzheimer’s disease,^16^ and schizophrenia.^17^ CD can be defined as a conversation between two or more people, where we communicate thoughts, ideas, and feelings to others in a cooperative interaction.^18^ As such, CD tasks often require he examiner and subject to engage in a conversation, where the examiner plays an active role, as one might in a normal conversation between two people. The natural format of this type of task makes it especially representative of patients’ daily interactions.

CD tasks are among the most difficult language tasks to administer, as they require the examiner to produce seemingly effortless spontaneous speech, while also engaging complex executive functioning. According to Van Dijk’s^19^ socio-cognitive model of discourse, the need for constant information updating renders this type of task especially difficult.^20^ Nevertheless, they allow for an in-depth investigation of CD, which is considered by the literature to be connected with several clinical and individual factors, including potential outcome indicators in acute illness.^11^^,^ 21

Age-related cognitive decline may influence a variety of language processes.^22^^,^ 23 According to Wingfield and Tun,^24^ age-related impairments may affect language comprehension skills and consequently, memory encoding. However, general linguistic knowledge is preserved and can be used as a strategy to improve performance in speech recognition and semantic memory tasks.^24^ Additionally, some of the individual differences in language abilities may be partially explained by the effects of aging on cognitive processes, such as divided attention, working and long-term memory. Impairments in these abilities may lead to age-related differences in performance on tasks which rely on these functions, such as CD.^24^^,^ 25

Longer speech segments and off-target verbosity are often observed in older adults.^7^^,^ 26 Their speech is often initially coherent and on topic, but over the course of a conversation, can turn to subject matters which are somewhat or even entirely unrelated to the matter at hand.^27^ Off-target verbosity can be observed in up to 22% of older adults,^28^ and elicit negative age-related stereotypes that question these individuals’ mental competence.^29^ Unconstrained rather than constrained discourse tasks have been found to be most effective in investigating verbosity.^30^

Age-related declines in syntactic processing were also discussed by Kemper,^31^ who hypothesized that a decrease in syntactic complexity such as subordinating clauses and coordinating phrases may be due to impairments in working memory or general cognitive slowing. No conclusions have yet been reached as to whether age-related language impairments are caused by a specific form of cognitive decline or a consequence of impairments in other cognitive functions. It has also been suggested that the repercussions of syntactic impairments may extend beyond oral speech and affect abilities such as written language and sentence imitation.^32^^,^ 33

In addition to their use as a standalone measure of language abilities, CD tasks can be incorporated into different aspects of the clinical setting. During history-taking, for instance, health professionals may take the opportunity to screen for impairments in communication skills. This information could later contribute to diagnostic, preventive or treatment interventions for discourse impairments produced by the process of aging.^1^

Though this topic has received increasing attention in the past decades, there is still a need to identify the discursive alterations associated with healthy ageing. Understanding changes in discourse behavior during typical aging will help to identify when a change may be larger than expected and therefore attributable to factors beyond aging, such as an underlying pathology. As far as we know, no other study has sought to investigate the main age-related changes in communicative behaviors during naturalistic conversation. Studies of healthy subjects are essential to ensure the accuracy of any future clinical data. A study comparing coherence between young-adults and older adults in different discourse modalities only found significant group differences when subjects were asked to speak about their weekend, a natural topic of conversation.^6^ However, the examiner was not an active participant in the task, and therefore could not assess the communicative interactions usually involved in everyday conversation.

Thus, the purpose of the present study was to compare the performance of young and older adults on a CD task developed to evaluate impairments in communicative behavior. It was hypothesized that younger adults would outperform older adults on measures of pragmatic cohesion and coherence, especially topic coherence, tangential and irrelevant information, word repetition and word search. The present study used the Complementary Procedure of Conversational Discourse Analysis (CPCDA),^8^ developed based on the CD task from the Montreal Communication Evaluation Battery.^34^^,^ 35 Performance was analyzed based on the items most commonly assessed in previous studies, as identified by a non-systematic review of the international literature. For more information about the analysis, see Pereira et al.^8^ The CD test involves a conversation between the subject and the examiner, where approximately two different topics are discussed over the course of at least four minutes, during which the researcher can identify and quantify any impaired communicative behaviors. The findings derived from this study will contribute to our comprehension of age-related differences in CD, and shed light on whether impaired communicative behaviors are specific to elderly participants, or simply more frequent in these individuals as compared to young adults.

## METHODS

### Ethical and data collection procedures

The data used in the present study was collected between 2015 and 2017, as part of two larger projects approved by the Research Ethics Committee of the Pontifical Catholic University of Rio Grande do Sul (PUCRS) under project numbers 11,077 and 657,955. Participation was voluntary and without financial compensation. All participants signed an informed consent form prior to study entry. All subjects were assessed by trained health professionals experienced in clinical neuropsychology, who conducted a comprehensive neuropsychological evaluation over the course of two to three sessions of approximately two hours each.

### Study sample

The data used in this investigation was drawn from the samples of two larger studies, which included a total of 250 participants, whose CD tasks had been previously analyzed and scored. The application of exclusion criteria specific to the present study led to the elimination of several individuals, resulting in a final sample of 54 young-adults and 41 older adults.

### Inclusion and exclusion criteria for the young adults sample

Young-adult subjects were recruited by convenience from university and community settings, and from personal referral. Participants were required to meet the following inclusion criteria: be native speakers of Brazilian Portuguese, 18 to 55 years of age and having at least four years of formal education. The exclusion criteria for the young adults sample were as follows: (1) current psychiatric disorders, as diagnosed by the DSM-IV Structured Clinical Interview for Axis I Disorders (SCID-I);^36^^,^ 37 (2) current or previous self-reported history of neurological disorders (stroke, tumor, epilepsy, brain injury); (3) current or previous history of self-reported substance abuse or dependence (alcohol, drugs and benzodiazepines).

### Inclusion and exclusion criteria for the older adults sample

Participants in the older adult sample were recruited from the community through radio and internet advertising, and included both individuals with general concerns about cognitive decline, as well as elderly people attending the Outpatient Dementia Clinic of the Hospital de Clínicas de Porto Alegre (HCPA). Participants were required to meet the following inclusion criteria: be native speakers of Brazilian Portuguese, at least 60 years old and have at least one year of formal education.

Participants who met any of the following criteria were excluded from the sample: (1) uncorrected sensory disturbances; (2) current self-reported psychiatric disorders that may interfere in their performance (depression, anxiety, post-traumatic stress disorders); (3) current or previous history of self-reported neurological disorders (stroke, tumor, epilepsy, brain injury); (4) current or previous history of self-reported substance abuse or dependence (alcohol, drugs and benzodiazepines); (5) presence of functional impairment as determined by the Activities of Daily Living Questionnaire, administered to a caregiver or family member;^38^ (6) IQ < 80 as determined by the WASI.^39^^,^ 40

### Instruments and procedures

#### Sample characterization procedures for young adults

Participants in the young-adults sample completed the following instruments:

(1) Sociocultural, medical and neuropsychological questionnaire for patients with traumatic brain injury (TBI).^41^ This instrument collects data on variables such as age, years of formal education, handedness (Edinburgh - Oldfield Handedness Inventory,^42^ adapted for use in Brazil, as described in the study of Brito, Brito, Paumgartten and Lins^43^), and socioeconomic status.^44^ The presence of general health conditions that may influence the results of future assessments, such as neurological, psychiatric, cardiac, and sensory problems is also investigated.^45^

(2) Structured Clinical Interview for DSM-IV Axis I Disorders (SCID-I).^36^^,^ 37 This interview aims to investigate the presence of Axis I mental disorders, as described by the Diagnostic and Statistical Manual of Mental Disorders (DSM-IV). Psychiatric evaluations were preferentially conducted with the participants themselves, though family members were asked to participate when participants were unable to complete the interview on their own.

#### Sample characterization procedures for older adults

Participants in the older adult group completed the following instruments:

(1) Clinical, medical and sociocultural questionnaire for elderly individuals. This is a semi-structured interview which can be used to screen for inclusion and exclusion criteria, and to collect sociodemographic and cultural data for sample characterization purposes. The questionnaire evaluates variables such as the frequency of writing and reading habits, socioeconomic status, age, sex, and years of formal education. Socioeconomic status was investigated using the Brazilian economic classification criteria,^46^ which provides a socioeconomic status score for the individuals based on ownership of several household items, the educational level of the family provider, and access to public services, such as running water and paved roads.

(2) Geriatric Anxiety Inventory (GAI).^47^ This scale evaluates any symptoms of anxiety experienced by the respondent in the previous week. Subjects are asked to indicate whether they agree or disagree with 20 phrases that describe common symptoms of anxiety. The scale provides a final score ranging from 0 to 20. In the present study, the cut-off adopted was ≥13.^48^

(3) Geriatric Depression Scale (GDS-15).^49^^,^ 50 This scale was developed to investigate depressive symptoms in the elderly. It contains 15 questions to be answered yes or no depending on whether the respondent feels it describes how they have felt over the past week. Scores range from 0 to 15 and can be classified as follows: no depression (0 to 5 points), mild-to-moderate depression (6 to 10 points) and severe depression (11 to 15 points).

(4) Activities of Daily Living Questionnaire.^38^^,^ 51 This questionnaire evaluates subjects’ functional capacity (self-care, social interaction and participation, intellectual activities, feeding ability) based on a caregiver interview. Its score ranges from 0 to 100, and can be used to classify functional impairment as mild (0-33), moderate,^34^^-^ 66 or severe (>66).

Additionally, older adults completed a comprehensive neuropsychological assessment battery which evaluated executive functions, attention, language, praxis, and memory. Further information on this assessment battery is available in an article published by Holz et al.^52^ Only subjects whose cognitive evaluations excluded the presence of cognitive impairment (i.e. z-scores greater than -1.5 on every cognitive domain) were included in the final sample.

#### Frequency of reading and writing

The frequency of reading and writing activities was examined for participants in both groups. This variable was investigated using an instrument which assesses how often the person reads (books, newspapers, magazines, and others) and writes (texts, messages, and others) (never (0), rarely (1), once a week (2), a few days a week (3), everyday (4)).^53^

#### Discourse assessment

All participants were evaluated using the CD task from the Montreal Communication Evaluation Battery - brief version.^35^^,^ 54 ^,^ 55 The analysis was conducted according to the CPCDA,^8^ a method of discourse analysis which evaluates impairments in communicative behaviors based on their frequency, with no maximum number of occurrences. As a result, the CPCDA yields a larger range of scores than the MAC Battery - brief version, whose scores only range between 0 and 2. As can be seen in Figure 1, the CPCDA is structured around 44 items, grouped according to the following discursive features: expression, pragmatics, cohesion, coherence, comprehension, non-verbal behaviors, emotional and linguistic prosody.

**Figure 1:** Complementary procedure for conversational discourse analysis

Discourse variable	Definition
**EXPRESSION**	
Total length of the audio file	Total length of the uncut audio recording (in seconds);
Total time analyzed	Length of the recording used for the analysis, including the discussion of both topics (in seconds);
Total Number of Words	Total number of words spoken by the participant, including repetitions, interjections and revisions;
Speed of speech	Total number of words divided by total speech time (in seconds);
Reaction time (RT)	Time taken to begin talking about the first topic (in seconds);
**COHERENCE**	
Coherence Topic 1(C1); Coherence Topic 2 (C2)	General measure of coherence scored on a scale of 1 to 4. A score of one (1) suggests the information provided by the participant was predominantly tangential and inaccurate, with the examiner having to guide the conversation and rely heavily on inferential comprehension to understand what the participant was attempting to convey. Two (2) points are attributed when the speech is somewhat related to the topic at hand, but includes inappropriate personal information, tangential comments or excessive emphasis on irrelevant elements of the conversation. The examiner may still rely on inferential processing to interpret what the participant is saying, but only at specific points in the dialogue, and not throughout the conversation as a whole. Three (3) points are given to participants whose speech is related to the topic at hand, and though it may contain some tangential or hypothetical information, it is still relevant to the themes under discussion. The examiner is able to understand what the participant says, though they may find their speech somewhat disorganized or insufficiently objective. Lastly, four (4) points are assigned when the speech produced by the participant is related to the topic at hand and contains enough information for the listener to understand its contents without relying on inferential processing (Carlomagno et al., 2011;Wright, Koutsoftas, Capilouto, & Fergadiotis, 2014);
**PRAGMATICS**	
Lacks verbal initiative (LI)	The participant cannot maintain the conversation without the aid of the examiner. They may answer the examiner's questions with a simple "yes" or "no", so that the dialogue resembles a question and answer session rather than a conversation;
Talks too much (TM)	The participant does not make pauses in their speech (long speech segments). The examiner is unable to ask questions or interrupt the conversation and may make unsuccessful attempts to interact with the participant. In these cases, the examiner is also unable to change the subject after two minutes or end the task after four;
Change of topic (CT)	The participant spontaneously introduces a new topic of conversation that is only tangentially or indirectly related to the topic at hand. The participant may or may not return to the original subject, with or without cues from the examiner. If 'CT' is maintained, it tends to progress to 'Does not follow the conversational topic (FCT)';
Does not return to the subject (DRS)	When the participant performs the subject change at first, then continues talking about the new subject until the speech time ends (or the examiner changes topic), without returning to the first prevailing topic prior to the exchange;
Returns to original topic with no help from the examiner (RTA)	The participant returns to the original subject after a Change of topic (CT), and does so of their own accord, with no feedback from the examiner;
Returns to original topic with help from the examiner (RTEH)	The participant returns to the original subject after a Change of topic (CT), but does so as a result of an external cue, such as a question asked by the examiner pertaining to the previous topic of discussion;
Changes topic due to examiner's interference (STEI)	The participant is inadvertently prompted to shift to an unrelated topic as a result of a comment made by the examiner. Given the naturalistic format of the task, this type of incident was expected to occur on occasion. However, participants were never penalized for it.
Interrupts the examiner (IS)	The participant interrupts the examiner mid-word or mid-sentence or talks over them. This behavior is usually indicative of impaired inhibitory control, and may prevent the examiner from contributing to the conversation, in addition to interfering with conversational turn-taking;
Inappropriate comments (IC)	The participant's comments make the examiner uncomfortable. Examples include the use of profanity or irrelevant and out of context information, such as speaking ill of a third party, whom the examiner does not know.
**COHESION**	
Abrupt interruption (AI)	The participant pauses or interrupts their speech abruptly. The listener may wait for them to complete the sentence, but this does not happen, and the content may be implied. This is often accompanied by non-verbal behaviors such as gesturing. This does not include instances where the participant stops talking, makes a longer pause, and continues discussing the current topic (which would be coded 'Inappropriate pauses - IP'), or when the participant interrupts a word or sentence in order to reformulate it before continuing to speak (in this case, 'Reformulates sentences or words - RSW') (adapted from Carlomagno, Giannotti, Vorano, & Marini, 2011);
Repeats word (RW)	All repetitions of words or phrases are coded, without exception. As such, the examiner does not need to determine whether the term was repeated in order to confirm an item of information or as a result of impaired self-monitoring (adapted from de Lira et al., 2011; Saling et al., 2014);
Repeats information (RI)	This is a variation of the previous item (RW), defined as any occasion where the participant repeats a given idea (i.e. information that has the same meaning or content). The repetition can be both immediate as well as interspersed throughout the dialogue (e.g. when a topic begins to be discussed, and again at the end of the conversation) (adapted from de Lira et al., 2011; Saling et al., 2014);
Inconsistent use of referential pronouns (IU)	This item is coded when the reference of a pronoun or the subject of a verb is ambiguous, unclear or incorrect (adapted from Galetto, Andreetta, Zettin & Marini, 2013). This behavior usually generates uncertainty in the examiner because they may not understand who or what the participant is actually talking about. For example, when talking about 'the dog' and 'the child' and soon after noting that "he is very sweet," the listener may not be sure who 'he' is if no contextual clues are provided;
Contradiction errors (CE)	The participant provides new information that contradicts what was previously said (Saling et al., 2014). If the participant notices the error and corrects themselves, the occurrence is coded as 'Reformulates sentences or words (RSW)' rather than a Contradiction Error (CE);
Relation errors (RE)	The participant presents new information that is not directly related to what was previously said (Saling et al., 2014). Such occurrences are unexpected, and may make the listener feel that the new information does not "fit" what was previously said, as if the sentences were not connected. Instances where the participant claims he will talk about a certain topic, but actually talks about a different - though related - subject, are also coded 'RE'. When an utterance is related to the overall theme of discussion, but is oddly placed, and requires additional inferential processing by the examiner, 'RE' may also be used. Likewise, if the participant notices the error and corrects what was said, the instance is scored 'RSW' rather than 'RE'. Lastly, it should be noted that, though this item is different from Change of topic ('CT'), one often precedes the other, so that an utterance coded 'RE' may be identified immediately before 'CT'.
Expresses ideas vaguely - confusing information (EVM- CI)	The participant's speech is unclear and imprecise. Phrases are syntactically correct but have little content. Though the participant does not speak too little, too slowly or has any difficulty finding words, their utterances are confusing and imprecise, with the examiner having to make significant efforts to interpret what was said. The speech may be tangential, and the listener may not understand the main points expressed by the participant;
Expresses ideas vaguely - insufficient information (EVM- II)	This is a variation of the previous item (EVM-CI). In this case, the participant does not give enough information on a given subject. The participant may talk too much (TM) while still providing little information, if their speech does not have enough elements to construct a logical story. This is different from 'Lacks verbal initiative (LI)', where the participant does not speak or engage in conversation;
Expresses ideas vaguely manner - sentence planning difficulties (EVM- DP)	This is a variation of the previous items (EVM-CI and EVM-II). In this case, participants have trouble organizing ideas or ordering facts in a story. The examiner may feel the speech is confusing, that the participant is talking in circles (mazes) or is having trouble finding the right word. This item is often accompanied by others such as 'Repeats word (RW)', 'Searches for words (SW)', 'Syllabic false starts (FS)' and 'Reformulates sentences or words (RSW)';
Grammatical errors - article use (EVM- IA)	Mistakes related to the agreement between articles and pronouns, nouns, adjectives, etc. For example, 'The man was walking/suddenly, she (instead of 'he') stopped' (Carlomagno, Giannotti, Vorano, & Marini, 2011);
Word-finding difficulties (SW)	The participant has difficulty retrieving words and may display anomia. These difficulties can often by identified by comments such as, 'that ... that thing... what do you call it?', or 'you know, the thing you use to do...'. Word-finding difficulties may also manifest as gaps in speech or prolonged vowels.
Paraphasia (PAR)	The participant exchanges one words or name for another. This category includes semantic, lexical and phonological paraphasia (adapted from Matsuoka et al., 2012). The occurrence can be followed by word-finding difficulties (SW), as in: 'Uh... (search for words)/ Use the pen to write' (when referring to the pencil);
Reformulates Sentences or Words (RSW)	Participant corrects themselves at the word/sentence level. When a correction is made at the syllable, it is coded as a 'Syllabic false start (FS)'. This includes the self-correction of speech errors... For instance, if the participant contradicts themselves (CE) but correct the error, the utterance will be coded 'RSW' rather than 'CE';
Syllabic false start (FS)	The participant abruptly interrupts their speech at the syllable level (either initial or final syllables). For example, 'two bo... / boys are prepar... / preparing for a game) (Galetto et al., 2013).
**COMPREHENSION**	
Does not understand what is said (UWS)	The participant does not understand questions or literal observations made by the examiner. Participants with this type of difficulty may be indifferent to questions asked by the examiner, or answer with unrelated remarks .
Does not maintain the conversational topic (FCT)	The participant performs a subject change and does not return to the original topic of conversation, either spontaneously or with the help of the examiner. This code also applies to instances where the participant forgets what they were saying mid-sentence. This item cannot be preceded by a question from the examiner, as it refers specifically to cases where the participant strays off topic of his own accord.
Does not understand indirect language (UIL)	Participant cannot understand indirect speech (e.g. E: 'Your phone is ringing' as an implicit suggestion for the participant to turn off his phone);
Does not understand figurative language (UFL)	Participant does not to understand figurative language, such as metaphors (e.g. 'Who wears the pants in the relationship?' or 'Are you a jack of all trades?').
Indifferent to jokes or light-hearted comments (SLC)	The participant does not respond or appears not to understand jokes or light-hearted comments made by the examiner. This may be accompanied by 'Does not understand what is said (UWS)';
Inconsistent or no eye contact (IEC)	The participant looks away from the examiner, or spends more time looking at objects or other people in the room.
Fixed facial expression (FFE)	The participant maintains the same facial expression throughout the entire conversation, and does not change it to match verbal expressions of emotion or variations in linguistic prosody;
Adapts poorly to subject change (APSC)	The participant has trouble changing topics halfway through the task. After the examiner signals the topic change, the participant may still want to finish a sentence or comment about the previous theme of conversation, or take longer to engage in the second topic of discussion (speech latency);
**LINGUISTIC AND EMOTIONAL PROSODY**	
Abnormal speech rate - increased (ASR-I)	Participant speaks abnormally fast. When this behavior is present, the patient receives the maximum score of one (1), regardless of how frequently the pattern occurred during the task. .
Has an abnormal speech rate - decreased (ASR-D)	Participant speaks abnormally slow. When this behavior is present, the patient receives the maximum score of one (1), regardless of how frequently the pattern occurred during the task.
Inappropriate pauses (IP)	The participant makes very long or frequent short pauses between words or ideas, changing the rhythm of the conversation. If the pauses occur while the patient is looking for a particular word, they are coded as 'Searches for words (SW)' rather than IP.
Speaks in monotone (MI)	Participant does not display any variations in linguistic or emotional prosody. The participant does not rely on prosodic cues for communication, speaking in a monotone or with restricted prosodic variability;
Abnormal linguistic prosody (ASIP)	Participant does not display the prosodic features associated with commands, statements and questions;
Does not respond to linguistic prosody (USIP)	Participant is unable to use prosodic cues to identify an utterance as a command, statement or question;
Abnormal emotional prosody (AEIP)	Participant does not display the prosodic features associated with emotions such as joy, sadness, anger or surprise;
Does not respond to emotional prosody (UEIP)	Participant is unable to use prosodic cues to identify emotions such as joy, sadness, anger or surprise in the speech of the examiner;
Number of metaphors provided by examiner (MP)	The number of metaphors used by the examiner during the conversation.

E: examiner; P: participant.

The CD task consists of a 4-minute conversation between the examiner and participant, where two familiar topics are discussed for approximately two minutes each. The participant is not given any prior information about the subject change or the duration of each part of the conversation; instead, the examiner introduces each topic in a natural conversational manner, using prompts such as “Tell me a little about your family (topic: family)”, and, after approximately two minutes, “Now about your work (topic: work): can you tell me a little about how it is or was?”. This task is intended to be as natural as possible, with the examiner asking relevant questions that facilitate the conversation and allow for the exchange of information. Additionally, the examiner must keep track of the two-minute time periods dedicated to the chosen topics, and in each insert a metaphorical expression such as “Who wears the pants in the relationship?” or “Are you a jack of all trades?”. The use of these metaphors allows the examiner to assess how the subject reacts to jokes or figurative language.

All interviews were audio-recorded and transcribed. Unintelligible utterances were excluded from the analysis. Each transcript was triple coded by the first author, with the first and last instances of coding separated by at least one week. During coding, the investigator was blind to participant groups. Each participant’s performance was scored according to the CPCDA, and results were written out on paper before being entered into a Microsoft Excel database. Separate files were created for each participant, and revised twice before being included in the final database.

In order to reduce the subjectivity of the CPCDA analysis, all items were scored as described in Figure 1. Poor quality recordings were excluded from the analyses, as were individuals with hearing impairments or fluency disorders. Given the variability in task duration as a result of individual differences in conversational engagement, only 2 minutes of each recording (with a 30-second tolerance interval) were analyzed for each participant. This procedure allowed the samples to be comparable in length. An example of the analysis procedure is shown in Appendix 1.

### Statistical analysis

Data were compiled and analyzed using SPSS Statistics version 20. Descriptive analyses were used to verify the frequency of impaired communicative behaviors in each group. Between-group comparisons of sociodemographic and clinical characteristics were conducted using Mann-Whitney tests. Chi-squared tests were used to evaluate between-group differences in the distribution of the following categorical discourse variables: number of questions asked before initiating the first topic of conversation, two-minute time limit exceeded, topic of conversation (family, work or leisure), presence of emotional alterations during the task, and examiner’s participation. Categorical variables were scored in order to describe the subjects’ discourse rather than diagnose impaired communicative behaviors. A multivariate analysis of covariance (MANCOVA) was conducted to compare the groups in terms of CD performance, with the frequency of reading and writing habits entered as a covariate. Results were considered significant at p≤0.05.

## Results

The sociodemographic characteristics of the two participant groups are shown in Table 1. Statistically significant group differences were identified in terms of age, as well as reading and writing habits. The number of years of formal education did not differ between participant groups.

**Table 1:** Sociodemographic characteristics of the sample.

	Young adults (n=54)	Older adults (n=41)	p
Age, years (mean±SD)	27.13(9.83)	69.68(6.76)	<0.001*
Education, years (mean±SD)	14.72(3.31)	14.32(5.61)	0.68
Socioeconomic status, score (mean±SD)	30.02(6.82)	34.50(11.16)	0.07
Reading and writing habits, score** (mean±SD)	17.93(4.83)	15.68(6.08)	0.04**
MMSE, score (mean±SD)	29.20(1.16)	28.12(2.24)	0.82

MMSE: Mini Mental State Examination.

* p≤0.05;

** parametric test.

Younger and older adults exhibited significant differences in 19 aspects (expression, pragmatic, cohesion, coherence, comprehension and prosodic and emotional linguistic) of discourse (Table 2). No such differences were identified in the remaining 23 items. In many of these cases, the majority of participants scored very close to zero, as was observed in the following variables: does not respond to linguistic prosody (USIP), does not understand indirect language (UIL), abnormal emotional prosody (AEIP), returns to original topic with no help from the examiner (RTA), indifferent to jokes or light-hearted comments (SLC), grammatical errors - article use (EVM-IA), does not understand what is said (UWS), contradiction errors (CE). Young adults scored lower than elderly individuals on all of these variables. Finally, six variables yielded a total score of zero, since the corresponding behaviors were not shown by any participants in the sample: changes topic due to examiner’s interference (STEI), inconsistent or no eye contact (IEC), adapts poorly to subject change (APSC), abnormal linguistic prosody (ASIP), Does not respond to emotional prosody (UEIP).

**Table 2:** Comparison of deviant communicative behaviors between healthy young adults and elderly participants.

		Young Adults (n=54)			Older Adults (n=41)		F	p	Cohen's d
		Mean(SD)	Min-Max		Mean(SD)	Min-Max			
Expression	Total length of the audio file	258.40(32.32)	183.00-415.00		298.80(93.35)	216.00-772.00	8.665	<0.001**	0.613
	Total time analyzed (topic 1)	128.46(15.84)	90.00-183.00		135.80(19.13)	86.00-189.00	4.1577	<0.001**	0.423
	Total time analyzed (topic 2)	126.22(17.57)	84.00-172.00		121.68(22.13)	71.00-166.00	1.227	0.271	0.231
	Total Time Analyzed	254.68(23.22)	183.00-315.00		257.48(23.29)	203.00-317.00	0.325	0.570	0.120
	Total Number of Words	457.26(134.89)	200-800		503.39(115.87)	228-780	3.036	0.085	0.363
	Speed of speech	1.78(0.45)	0.86-2.75		1.95(0.40)	0.92-2.74	3.331	0.071	0.396
	Reaction time (RT)	4.92(3.22)	0.00-15.00		5.26(7.28)	0.00-45.00	0.088	0.767	0.063
Coherence	Coherence Topic 1 (C1)	3.87 (0.34)			3.41 (0.67)		19.005	<0.001**	0.904
	Coherence Topic2 (C2)	3.85 (0.36)			3.37 (0.73)		18.618	<0.001**	0.872
Pragmatics	Lacks verbal initiative (LI)	1.19(1.89)	0-9		0.32(0.90)	0-5	7.271	<0.001**	0.563
	Talks too much (TM)	0.17(0.86)	0-6		0.93(1.23)	0-4	12.354	<0.001**	0.734
	Change of topic (CT)	0.06(0.30)	0-2		0.73(0.97)	0-3	23.712	<0.001**	0.992
	Does not return to the subject (DRS)	0.00(0.00)	0-0		0.32(0.65)	0 -2	12.820	<0.001**	0.751
	Returns to original topic with no help from examiner (RTA)	0.02(0.13)	0-1		0.05(0.21)	0 -1	0.700	0.405	0.177
	Returns to original topic with help from the examiner (RTEH)	0.04(0.27)	0-2		0.37(0.58)	0-2	14.196	<0.001**	0.765
	Interrupts the examiner (IS)	1.09(1.27)	0-6		1.41(1.77)	0-8	1.048	0.309	0.213
	Inappropriate comments (IC)	0.15(0.73)	0-5		0.15(0.35)	0-1	0.000	0.991	0.000
Cohesion	Abrupt interruption (AI)	1.00(1.06)	0-4		1.27(1.04)	0-3	1.489	0.225	0.257
	Repeats word (RW)	16.61(6.51)	4-30		15.39(6.05)	6-29	0.875	0.352	0.193
	Repeats information (RI)	5.87(3.76)	0-16		6.51(3.10)	1-13	0.766	0.384	0.183
	Inconsistent use of referential pronouns (IU)	0.11(0.37)	0-2		0.54(0.92)	0-5	9.374	<0.001**	0.647
	Contradiction errors (CE)	0.07(0.26)	0-1		0.27(0.80)	0-4	2.710	0.103	0.357
	Relation errors (RE)	0.20(0.40)	0-1		1.24(1.42)	0-5	26.043	<0.001**	1.062
	Repeats the last words said by the examiner (RSE)	1.63(1.87)	0-7		1.56(1.65)	0-7	0.031	0.862	0.039
	Expresses ideas vaguely-confusing information (EVM-CI)	0.06(0.23)	0-1		0.90(1.17)	0-4	26.298	<0.001**	1.068
	Expresses ideas vaguely-insufficient information (EVM-II)	0.63(1.08)	0-5		0.29(0.75)	0-4	3.052	0.084	0.357
	Expresses ideas vaguely-sentence planning difficulties (EVM-DP)	0.19(0.43)	0-2		0.59(0.70)	0-2	11.449	<0.001**	0.711
	Grammatical errors-article use (EVM-IA)	0.02(0.13)	0-1		0.02(0.15)	0-1	0.032	0.859	0.000
	Word-finding difficulties (SW)	12.7(6.25)	4-31		9.98(4.15)	2-16	6.021	0.016*	0.499
	Paraphasia (PAR)	0.00(0.00)	0-0		0.15(4.22)	0-2	6.495	0.012*	0.054
	Reformulates Sentences or Words (RSW)	3.8(2.74)	0-12		5.24(2.71)	0-13	6.458	0.013*	0.528
	Syllabic false start (FS)	2.93(2.46)	0-12		3.10(2.55)	0-10	0.102	0.750	0.068
Comprehension	Does not understand what is said (UWS)	0.04(0.19)	0-1		0.15(0.52)	0-3	1.963	0.165	0.297
	Does not maintain the conversational topic (FCT)	0.00(0.00)	0-0		0.22(0.52)	0-2	9.405	<0.001**	0.645
	Does not understand indirect language (UIL)	0.00(0.00)	0-0		0.02(0.15)	0-1	1.298	0.258	0.203
	Does not understand figurative language (UFL)	0.07(0.26)	0-1		0.27(0.54)	0-2	5.185	0.025*	0.494
	Indifferent to jokes or light-hearted comments (SLC)	0.02(0.13)	0-1		0.05(0.21)	0-1	0.689	0.409	0.68
Linguistic and emotional prosody	Abnormal speech rate-increased (ASR-I)	0.44(0.79)	0-2		0.88(0.98)	0-2	5.740	0.019*	0.502
	Abnormal speech rate-decreased (ASR-D)	0.17(0.50)	0-2		0.27(0.67)	0-2	0.693	0.407	0.173
	Inappropriate pauses (IP)	0.41(0.74)	0-3		0.56(0.92)	0-4	0.791	0.376	0.182
	Speaks in monotone (MI)	0.13(0.47)	0-2		0.27(0.67)	0-2	1.361	0.246	0.248
	Does not respond to linguistic prosody (USIP)	0.00(0.00)	0-0		0.10(0.49)	0-3	2.139	0.147	0.311
	Abnormal emotional prosody (AEIP)	0.00(0.00)	0-0		0.05(0.31)	0-2	1.295	0.258	0.246

* p≤0.05;

** p≤0.001 "-" the discourse variables: Changes topic due to examiner's interference (STEI), inconsistent or no eye contact (IEC), Adapts poorly to subject change (APSC), abnormal linguistic prosody (ASIP), Does not respond to emotional prosody (UEIP) were not included in the table because no participants obtained positive scores on these variables.

Young adults outperformed their elderly counterparts on all but two variables, namely, lacks verbal initiative (LI) and has word-finding difficulties (SW). Although 43.9% (n=18) of elderly participants asked questions before initiating the first topic of conversation, as compared to only 31.5% (n=17) of young-adults, this difference was not statistically significant. Prior to the second topic of conversation, questions were asked by 12.2% (n=5) of elderly participants and 3.7% (n=2) of young-adults. The frequency with which participants exceeded the optimal (two minutes) time period for each topic of conversation was also similar between participant groups. This phenomenon was observed in 68.3% (n=28) of elderly participants and 61.1% (n=33) of young adults in the first topic of conversation, and 51.2% (n=21) of elderly subjects and 61.1% (n=33) of young adults in the second topic of conversation. As a result, 87.8% (n=36) of older adults and 68.5% (n=37) of younger participants exceeded the four-minute time limit for the task as a whole.

In the majority of cases, ‘family’ was the first topic discussed by both elderly (97.6%; n=40) and young participants (96.3%; n=52). The second subject in most cases was ‘work’, as observed in 80.5%; n=33 of older adults and 79.6% (n=43) of young-adults, followed by ‘leisure’ (older adults: 17.1%; n=7; younger adults: 16.7%; n=9). Emotional behaviors such as crying or irritability were displayed by 9.8% (n=4) of elderly participants, and no younger adults. The examiner played an active role in the conversation for 87.8% (n=36) of the elderly sample and 96.3% (n=52) of younger adults.

## DISCUSSION

The main purpose of this article was to compare the performance of young and older adults on a CD task where impaired communicative behaviors were evaluated using the CPCDA. The two groups differed on 19 items of the CPCDA pertaining to expression, pragmatics, cohesion, coherence, comprehension, emotional and linguistic prosody. The findings did not confirm the hypothesis that group differences would be found in behaviors associated with speech planning and self-monitoring, such as: repeats words (RW), repeats information (RI), syllabic false starts (FS), abrupt interruptions (AI) and repeats the last words said by the examiner (RSE). The present findings revealed that both groups obtained similar scores for these variables.

One possible explanation for the findings is that these behaviors are a normal part of speech for healthy individuals across all age groups. Repetition and abnormal word sequencing, for instance, may be used as a way to emphasize ideas. As far as we know, no previous study has compared these particular behaviors between younger and older adults. However, Dijkstra, Bourgeois, Burgio and Allen^56^ have identified an important difference between errors in cohesion, coherence and conciseness - referred to as discourse-building features - and alterations such as revisions, syllabic false starts, interruptions, and repetitions. According to the authors, the latter are common in more naturalistic situations, which may explain their similar frequency in both groups of the present study.

Repetitions and extensions may also be used as strategies for accessing and/or retrieving the next word within a sentence. In this situation, the repeated word is used as a retrieval cue for a related term we are having trouble accessing.^57^ Yet repeating a word or phrase may not always help an individual recall a particular word. When this strategy is overused or ineffective, it may become distracting to the listener, and compromise their understanding of the conversational content. Future studies may wish to investigate this type of behavior in narrower age groups in order to gain a deeper understanding of its occurrence in healthy individuals, especially since the available literature offers no definitive conclusions on the topic.

Additionally, no differences were found between groups in terms of the total number of words uttered or the speed of speech. Wright, Capilouto, Srinivasan and Fergadiotis^58^ found significant differences between healthy younger and elderly subjects in a scene description task. The results indicated that elderly participants needed more words than young adults to transmit the same message. However, this difference may have been observed due to the type of discourse assessed. In a conversational situation with no predetermined content parameters, like that observed in the present study, younger and older adults may not differ in this regard.

Another expected finding was a higher initial reaction time (speech latency) among older adults due to general age-related slowing.^59^^,^ 60 However, this was not the case. Though this finding does not negate the idea of age-related slowing, it is possible that the effects of this phenomenon vary across cognitive domains and subdomains, and are influenced by other cognitive abilities, such as working memory.^61^

Contrary to present findings, previous studies have found younger adults to be faster than elderly participants on all variables and conditions of a narrative discourse task.^3^ Differences between younger and older adults in tasks with high verbal complexity and low visuospatial complexity have also been found.^61^ However, it is important to emphasize that the familiar and spontaneous nature of the conversational discourse task facilitates access to information. Moreover, it relies on information which participants can access through their semantic memory, a cognitive ability that tends to improve over the course of normal aging.^59^

The absence of group differences in speech latency in the present study is likely due to the type of discourse evaluated in the present investigation. According to Davis and Guendouzi,^62^ Dijkstra, Bourgeois, Allen and Burgio^63^ and Knitsch and Van Dijk,^20^ performance on dynamic speech tasks often benefits from pre-established mental models and analogical representations that facilitate the analysis and interpretation of the information received. This may be the case in measures of narrative discourse, for instance, where subjects’ performance may benefit from the linguistic context of pre-established mental models of story-telling.^64^ The CD task, on the other hand, does not have a pre-established structure, and the speed with which a mental model can be accessed therefore has no influence on performance.

Another interesting finding was that young adults showed less conversational engagement than older participants, as evidenced by the higher frequency of low verbal initiative in the former as compared to the latter. Although young adults produced fewer words per minute and had more word-finding difficulties than older adults, they were more coherent than older individuals for both topics of conversation. This may be because young people are less used to talking about their personal lives with strangers and/or did not realize this was part of the evaluation. Elderly individuals, on the other hand, speak far more often about their lives, though they are more likely than young adults to provide tangential and less accurate information about these topics. The communicative behaviors of elderly individuals differ from those of younger adults in several respects: they argue more often, have different communicative goals, emphasize the specific description of some events over others, and have more difficulty being direct and objective.^30^ The current literature often attributes these phenomena to a lack of verbal inhibitory control.^26^ The low verbal initiative and word-finding difficulties displayed by younger adults may explain why, results contrary to previous findings,^65^ the frequency of a behavior described as “expresses his/her ideas in a vague manner - insufficient information” was higher among younger than older adults in the present investigation.

The variables for which the young adults outperformed older participants will be discussed according to the linguistic component evaluated (i.e. expression, coherence, pragmatics, cohesion, comprehension, and linguistic or emotional prosody). As previously stated, findings regarding linguistic expression did not confirm our expectations. Elderly participants did score higher than younger individuals for the variables “total length of the audio file” and “ total time analyzed for topic 1”, which at first glance appears to confirm the idea that elderly individuals are more loquacious than their younger counterparts. However, we must understand the length of the audio file may have been influenced by several other variables, including the repetition of ideas, number of questions and interruptions by the examiner, time spent in silence while the participant plans their next utterance, and even slowed speech. As such, the duration of the task or of its subsections cannot by itself support any claims regarding participants’ communicative behaviors, and must necessarily be supplemented by the analysis of complementary variables.

The most important measures of discourse coherence in the CD task are “coherence in topic 1” and “coherence in topic 2”, which provide a general idea of how speech is organized over time and the extent to which participants’ responses relate to the questions posed by the examiner. In the present study, young adults outperformed older participants on both measures of coherence. Previous studies have also reported differences between age groups on similar measures of coherence, even when examining discourse modalities which differ from that evaluated in the present study.^6^^,^ 66 The combined findings of this and previous studies suggest that elderly individuals provide more concrete and less accurate information than younger adults on the topics discussed.

In addition to investigating these variables further, future studies should examine their association with other cognitive abilities, such as speech speed control and executive attention.

The performance of elderly subjects on the pragmatic aspects of language was exactly as expected. The presence of significant group differences on pragmatic variables demonstrated that elderly individuals may have difficulty putting themselves in the examiner’s place, as evidenced by an inability to engage in conversational turn-taking (TM), difficulty maintaining the topic of conversation (CT) or returning to the original subject after a topic change (DRS). In many cases, elderly participants were only able return to the current topic of conversation with the help of cues from the examiner (RTEH). These results confirm previous findings regarding functional impairments in speech associated with alterations in verbal inhibitory control. The ability to stay on topic is also influenced by discursive coherence.^67^^,^ 68 Incoherent and tangential utterances, as well as disorganized speech, were all more common among older adults, which led them to stray from the topic of conversation more often than younger individuals.^67^

Cohesion is also related to, and a direct influence on, discourse coherence. In this study, elderly individuals often displayed inconsistencies in referential cohesion. As a result, in order to comprehend what is said, the other interlocutors must closely monitor the linguistic content produced by the elderly individual, who is unable to do so on their own. Outsourcing the organization and planning of speech allows the interlocutor to determine the topics discussed by the participant based on their own understanding of the referential expressions used. This may be problematic, since referential speech is often ambiguous, and the listener may not always be able to correctly interpret what the elderly individual is attempting to convey.^69^ This issue is aggravated by the fact that elderly adults have difficulty processing speech structures and selecting appropriate referential terms. The excessive use of pronouns by elderly individuals may also be associated with alterations in other cognitive abilities, such as memory span.^70^

Communicative behaviors such as disconnected utterances, confusing language and poor sentence planning are also indicative of impairments in functional language skills. All of these behaviors are associated with the need to organize, plan and maintain information “online” for the purpose of discursive processing. Alterations in these behaviors interfere with coherence, since they may lead to the insertion of unrelated phrases in the discussion of a particular topic, as reported by Marini, Boewe, Caltagirone and Carlomagno.^71^ Behaviors related to lexical-semantic access, such as paraphasias and the reformulation of sentences or words support the idea that difficulties in lexical access are common among the elderly (71). Lexical, phonological and morphological access are considered features of microlinguistic processing, all of which appear to worsen with aging.^67^

Finally, comprehension was evaluated through two main behaviors: inability to maintain the topic of conversation and to understand figurative language. Comprehension is thought as the most basic component of communication, and a major prerequisite for social participation. Many studies have been carried out using written comprehension and oral narrative discourse tasks, and found that elderly individuals tend to make more mistakes during these activities relative to younger subjects.^72^^-^ 74 While younger adults focus on the text itself and its microstructure, older individuals are more likely to cling to the general idea of the text, suggesting difficulty in understanding nuance and/or specific utterances during a discourse task.^75^^,^ 76

Successful comprehension is what allows participants to extract the most important information from a conversation. Across all forms of communication, comprehension occurs sequentially, linking new information to what was already known from an earlier point in time (mental models).^20^ Yet unlike studies involving writing tasks, some investigations found no differences between age groups with regards to the understanding of verbal material, and questioned the extent to which age influences the understanding of mental models.^77^^,^ 78 Nevertheless, comprehension is influenced by both cognitive and sensory (e.g. auditory loss) impairment.^79^^,^ 80 As such, in addition to investigating comprehension in connection with different aspects of cognition, future studies may also want to investigate the association between these variables and measures of sensory alterations such as hearing loss.

An inability to maintain the topic of conversation has already been described in previous studies of elderly individuals.^7^^,^ 21 ^,^ 81 This behavior appears to be directly influenced by changes in other cognitive abilities such as attention, memory and executive processing.^10^^,^ 27 ^,^ 81

Lastly, as far as linguistic and emotional prosody are concerned, no studies have included these features in evaluations of verbal discourse. However, studies have identified changes in speech production as a result of the impact of aging on anatomical structures and functions, motor control of speech, breathing patterns, phonation, resonance, articulation, fluency^79^ and decreased fundamental frequency.^82^ The use of speech analysis software may help provide a more objective assessment of prosody and contribute to studies on the relationship between this variable and mood changes such as depression and apathy, as well as speech speed.

The accurate assessment of discursive behavior may be very useful for the early diagnosis of cognitive decline. Unfortunately, due to the time-consuming nature of these analyses, they are rarely used in clinical settings. Future studies should analyze these variables and compare them between healthy older adults, patients with mild cognitive impairment and individuals with mild dementia, in order to contribute to the planning of discourse intervention programs.

In conclusion, the present study compared young and older adults on a measure of conversational discourse using the CPCDA. The results showed an interesting profile of communicative behaviors that seems to be found in healthy individuals regardless of age. This profile is characterized by the repetition of words and information units, syllabic false starts, abrupt interruptions and repeating the last thing said by the examiner. Additionally, variables concerning expression, coherence, pragmatics, cohesion, comprehension, and linguistic and emotional prosody were more likely to display alterations in older adults.

The present study has some limitations that should be considered. First, to our knowledge, no prior study has evaluated CD in such a detailed way. In general, when this type of ability is examined, fewer variables are analyzed and comparisons are made between clinical groups rather than healthy participants. The lack of previous studies using the CPCDA and the complexity of this type of assessment must also be noted. However, this procedure did allow for the identification of communicative alterations similar to those reported in previous studies, including those involving clinical groups. Thus, even though the present study applied the CPCDA to a population of healthy adults, the present findings demonstrate that this may be a useful tool to identify communicative patterns like those observed in previous studies.

The present findings emphasize the importance of including assessments of CD when evaluating communicative profiles, precisely due to the naturalistic and ecological format of the task, in addition to its low cost. The procedure used in the present study needs to be replicated in clinical populations, such as individuals with neurological impairments. Furthermore, it may be interesting to identify which variables may be most relevant in this type of assessment and most closely related to impairments in cognitive abilities such as attention, memory and executive functions, so that these can be included in screening measures for clinical use.

## APPENDIX 1 Example of the analysis procedure

Subtitle

*Italic Font and [ ]: [comments about the score]*

**Bold Font: the sentence in the text that was scored**

Female, 87 years, 5 years of formal education, this patient was a control in database, without any deficits in the battery test

*[the patient presents a fast speech, thus receiving the score: ASR-I]*

*[the subjet one “family” starts]*

...

P: This is my drama.

E: So tell me a little about your family.

P: I had three children, I lost the eldest during a robbery **but, regardless of that, I was married for thirty years** *[Here we expected her to speak about her daughter, so she receives the score: “RE” and “EVM- CI” because the information does not seem clear like: “I continued this vain”]*. In the last seven years, my husband worked outside the home, he was always so **vain** and I **continued** *[we think this is not the best word for it so, receives the score “PAR”]* this **vain** *[receives the score “RW”]*

P: And **there** *[until this moment, we don´t know where it is“there”, so receives the score “IU”]*, then he was sent to the best hotel in the city and then **he** found out that **he** *[receives the score “RW”]* was the “the best thing since sliced bread” [*she wants to say: “he considers himself the best person”*]. When he came home, it was like hell. **He** *[receives the score “RW”]* had always, let’s **say...** *[makes a short pause to search the word, so receives the score “SW”]*, **cultivated** *[we think this is not the best word for it, so receives the score “PAR”]* his beauty. I never payed attention to that.

**He encouraged that**... [she repeats the mean idea about “cultivated this aspect”, so receives the score “RI”]. I worked **for a tailor** *[here we don´t expected this information, so receives the score “RE” and “CT” because from here she doesn´t continue talking in the same subject].* I wanted to see **so so** *[receives the score “FS”]* his family would accept me. That in my husband’s **family** *[receives the score “RW”]*, male child is **family’s** property *[receives the score “RW”]*, **no one touches** *[repeats the mean ideia so receives the score “RI”]*. The women have to find a man to sustain them... until soon .. but I married the oldest one .. **then**...*[she stops the sentence abruptaly therefore receives “AI”] [another* aspect is that she can´t return at the first subject therefore punctuating “DRS” and “FCT”][Lastly, in the entire sentece we punctuating “EVM- CI” and“EVM- DP”].

E: **But did you get along with him?** *[the examiner, realizing that she wouldn´t return to the subject “family” tries to lead the conversation, therefore punctuating “RTEH”]*

P: **I** *[makes a short pause to search the word, so receives the score “SW”]* was seeking to get along… **get inside of**… *[she stops the sentence abruptaly therefore receives “AI”]* then when he came then it was a very difficult situation. **I** was older and less able to work because I always worked a lot .. the children were studying, finishing college and **everything. I always was a mother, father, friend, teacher, I was all together and there the problems began** *[she doesn´t answer the question and add a new information, therefore punctuating “RE” and “RI”]* so he decided to go on adventures... all right ... I used to pretend I did not see not to be bothered, but he got to the point he wanted to bring to live inside our house a cousin of mine who had an affair with him *[pelo fato da paciente não deixar o examinador par- ticipar tanto ela é pontuada nesse momento como “FM]*.

I did not accept it. **I did not accept** *[receives the score “RW”]* fight here fight there *[receives the score “RW”]*... until at one moment he was desperate because she was making his life a living hell... he assaulted me... because he wanted me to leave only with the clothes on my body that it was fine and his sisters hated me later because they said that I had no right to go to court against him *[this type of information was not expected at that time, therefore punctuating “RE” and “CT”]*

E: So it was kind of a troublesome marriage and how was your job? *[the examiner, realizing that she wouldn´t return to the subject “family” tries to lead the conversation, therefore punctuating “RTEH”]*

(2: 18) *[end of the subject one]*

*[we emphasize that the evaluator did not participate a lot in this conversation. He could have made more questions] [cohesion: score for this subject = 2]*

Subtitle

*Italic Font and [ ]: [comments about the score]*

**Bold Font: the sentence in the text that was scored**

Male, 82 years, 16 years of formal education, this patient was a control in database, without any deficits in the battery test

*[the patient presents a fast speech, thus receiving the score: ASR-I]*

*[the subject one “family” ends and initiate the subject “work”]*

...

E: Yes, and tell me... you are retired now, right? Tell me a little about how was your job...

P: Well, my job **is**... *[makes a short pause to search the word, so receives the score “SW”]* **is more**... *[makes a short pause to search the word, so receives the score “SW”]* **is** *[makes a short pause to search the word, so receives the score “SW”]...* it is more about taking care of of *[does syllabic false start “FS”]* business al- though… **bi.. pa...** *[reformulates Sentences or Words“RSW”]* the bills, shoppings and… schedule the appoin- tements… stuff like that, huh?... it’s me who does everything. The wife she likes **to to** *[makes a short pause to search the word, so receives the score “SW”]* work at home

E: Yes

P: she wants to go out sometimes and go for a walk, to go out *[repeated word directly receives the score* “RW”] [this first part the patient receives “EVM- CI”]. But it is very unusual… *[here the examiner tries to ask a question]* to go to the bank… she already been there several times with me to **learn** *[repeated word di- rectly receives the score “RW”]* how to deal with the *[not abble to understand]* but she does not learn because stries a hundred times and then forget *[here we don´t expected this information, so receives the score “RE”]* [the whole sentence is confuse, receives “EVM- DP”]; [The participant does not understand questions or literal observations made by the examiner, receives “UWS”]; [The examiner is not able to ask questions or *interrupt the conversation, receives “TM”]*

E: So, currently this is your job, huh?

P: This is my job. *[repeats the mean ideia so receives the score “RI”]*

E: But formerly, what was your job?

P: **Formerly?** [here, the patient repeats the last thing said by the examiner and so it scored “RSE’] No… when I worked? **No… so… so…** *[makes a short pause to search the word, so receives the score “SW”]* I **worked** *[repeated word directly receives the score “RW”]* a lot... Look… I worked… *[here he interrupts his speech in an abrupt way, receives the score “AI”]* there was some occasions which we had lot of helpers... there *[repeat- ed word directly receives the score “RW”]* was some occasions that I was just by my self... other times one or two was not enough we worked a lot. *[the whole sentece is confuse receives “EVM- CI”]*

E: What exactly did you do? P: Uhm?

E: What did you do at your work?

P: Nowadays? *[The participant does not understand questions made by the examiner, receives “UWS”]* Now- adays *[does syllabic false start “FS”]* I’m part of the fiscal category. But I never was fiscal *[repeated word directly receives the score “RW”]*, I was part of the tax revenues. [here the participant presents difficulty in organizing the ideas or sequence of the facts in a story, receives “EVM-DP”]

E: But what was your job? *[for the third time the examiner tries to conduce the conversation]*

P: I... *[makes a short pause to search the word, so receives the score “SW”]* well... receive the money. E: Hmmm, yes!

P: Nowadays, money *[repeated word directly receives the score “RW”]* goes all the way to the bank.. but in my time.. all..all of [here the patient speaks two different words which is difficult to translate, but he receives *“RSW”]* the money went to the extortionate [tax office responsible for collecting taxes] so I paid the city functionalism of of *[does syllabic false start “FS”] [makes a short pause to search the word, so receives the score “SW”]* all of them, since the judge till the school servant… I paid *[repeats the mean ideia so receives the score “RI”]* it all through them, received the money from the taxes *[makes a short pause to search the word, so receives the score “SW”]* and then, daily, the report.

**E: Yes**

**P: The report** *[repeated word directly receives the score “RW”]*and…*[makes a short pause to search the word, so receives the score “SW”]*the money left over I sent to Porto Alegre… When there was a lack the state **ordered** *[repeated word directly receives the score “RW”]* to complement…

E: That´s good!.. Yes!

P: That’s it… well… I always dealt with money.

*[cohesion: score for this subject=2]*

*[We emphasize that was done a literal translation from Portuguese to English. We tried to be the more trust- worthy as possible. It is important to point that the conversational discourse transcription, resultant from translation to English, became confuse because patient´s impairment (the discourse was also confusing in the original version)]*

## References

[1] Ska B, Scherer LC, Flôres OC, Oliveira CR de, Netto TM, Fonseca RP (2009). Theoretical, behavioral and neuroimage evidence on discourse processing aging. Psychol Neurosci.

[2] Kemper S, Anagnopoulos C (1989). Language and Aging. Annu Rev Appl Linguist.

[3] Martin CO, Pontbriand-Drolet S, Daoust V, Yamga E, Amiri M, Hübner LC (2018). Narrative discourse in young and older adults: Behavioral and NIRS analyses. Front Aging Neurosci.

[4] Whitworth A, Claessen M, Leitão S, Webster J (2015). Beyond narrative: Is there an implicit structure to the way in which adults organise their discourse?. Clin Linguist Phonetics.

[5] Menon DK, Schwab K, Wright DW, Maas AI (2010). Position statement: Definition of traumatic brain injury. Arch Phys Med Rehabil.

[6] Wright HH, Koutsoftas AD, Capilouto GJ, Fergadiotis G (2014). Global coherence in younger and older adults: Influence of cognitive processes and discourse type. Aging, Neuropsychol Cogn.

[7] Arbuckle TY, Pushkar D, Bourgeois S, Bonneville L (2004). Off-target verbosity, everyday competence, and subjective well-being. Gerontology.

[8] Pereira N, Hübner L, Casarin F, Zimmermann N, Ferré P, Joanette Y (2015). Procedimento complementar de análise do discurso conversacional por frequência de comportamentos comunicativos desviantes Complementary procedure of conversational discourse analysis. Rev Neuropsicol Latinoam.

[9] Glosser G, Brownell H, Joanette Y, Brownell HH, Joanette Y (1993). Discourse production patterns in neurologically impaired and aged populations. Narrative discourse in neurologically impaired and normal aging adults.

[10] Rogalski Y, Altmann LJP, Plummer-D'Amato P.Behrman AL.Marsiske M (2010). Discourse coherence and cognition after stroke: A dual task study. J Commun Disord.

[11] Coelho CA, Liles BZ, Duffy RJ (1995). Impairments of discourse abilities and executive functions in traumatically brain-injured adults. Brain Inj.

[12] Pistono A, Jucla M, Barbeau EJ, Saint-Aubert L, Lemesle B, Calvet B (2016). Pauses During Autobiographical Discourse Reflect Episodic Memory Processes in Early Alzheimer's Disease. J Alzheimer's Dis.

[13] Shadden BB, Burnette RB, Eikenberry BR, DiBrezzo R (1991). All discourse tasks are not created equal. Clin aphasiology.

[14] Marini A, Galetto V, Zampieri E, Vorano L, Zettin M, Carlomagno S (2011). Narrative language in traumatic brain injury. Neuropsychologia.

[15] Strauss Hough M, Barrow I (2003). Descriptive discourse abilities of traumatic brain-injured adults. Aphasiology.

[16] Brandão L, Parente MA, Peña-Casanova J (2008). Turnos E Atos De Fala Do Interlocutor De Pessoas. ReVEL.

[17] Rabagliati H, Delaney-Busch N, Snedeker J, Kuperberg G (2018). Spared bottom-up but impaired top-down interactive effects during naturalistic language processing in schizophrenia: evidence from the visual-world paradigm. Psychol Med.

[18] McDonald S, Togher L, Code C, McDonald S, Togher L, Code C (2013). Social and Communication Disorders Following Traumatic Brain Injury. Social Communication Disorders Following Traumatic Brain Injury.

[19] Van Dijk TA (2012). A note on epistemics and discourse analysis. Br J Soc Psychol.

[20] Kintsch W, Van Dijk TA (1978). Toward a Model of Text Comprehension and Production. Psychol Rev.

[21] LeBlanc J, de Guise E, Champoux M-C, Couturier C, Lamoureux J, Marcoux J (2014). Early conversational discourse abilities following traumatic brain injury: An acute predictive study. Brain Inj.

[22] Orange JB, Purves B (1996). onversational Discourse and Cognitive Impairment: Implications for Alzheimer's Disease. J Speech-Language, Pathol Audiol.

[23] Van Dyke JA (2012). The role of memory in language and communication. Cognition and Acquired Language Disorders.

[24] Wingfield A, Tun PA (2001). Spoken Language Comprehension in Older Adults: Interactions between Sensory and Cognitive Change in Normal Aging. Semin Hear.

[25] Connor LT (2001). Memory in Old Age: Patterns of Decline and Preservation. Semin Speech Lang.

[26] Yin S, Peng H (2016). The Role of Inhibition in Age-related Off-Topic Verbosity: Not Access but Deletion and Restraint Functions. Front Psychol.

[27] Arbuckle TY, Gold DP (1993). Axging, Inhibition, and Verbosity. J Gerontol.

[28] Gold DP, Arbuckle TY, Andres D, Hummert ML, Wiemann JM, Nussbaum JF (1994). Verbosity in older adults. Interpersonal communication in older adulthood: Interdisciplinary theory and research.

[29] Ruscher JB, Hurley MM (2000). Off-Target Verbosity Evokes Negative Stereotypes of Older Adults. J Lang Soc Psychol.

[30] James LE, Burke DM, Austin A, Hulme E (1998). Production and perception of "verbosity" in younger and older adults. Psychol Aging.

[31] Kemper S (1987). Life-span Changes in Syntactic Complexity. J Gerontol.

[32] Kynette D, Kemper S (1986). Aging and the loss of grammatical forms: a cross-sectional study of language performance. Lang Commun.

[33] Kemper S (1986). Imitation of complex syntactic constructions by elderly adults. Appl Psycholinguist.

[34] Fonseca RP, Parente MA de MP, Cote H, Ska B, Joanette Y (2008). Bateria Montreal de Avaliação da Comunicação - Bateria MAC.

[35] Joanette Y, Coté H, Ska B (2004). Protocole MEC - Protocole Montreál D'Évaluation de La Communication.

[36] First MB et, Spitzer RL, Gibbon M, Williams JBW (2002). Structured Clinical Interview for DSM-IV-TR Axis I Disorders, Research Version, Non-patient Edition. for DSMIV.

[37] Del-Ben CM, Vilela JAA, Crippa JA de S, Hallak JEC, Labate CM, Zuardi AW (2001). Confiabilidade da "Entrevista Clínica Estruturada para o DSM-IV - Versão Clínica" traduzida para o português. Rev Bras Psiquiatr.

[38] Medeiros M, Guerra R (2009). Tradução, adaptação cultural e análise das propriedades psicométricas do Activities of Daily Living Questionnaire (ADLQ) para avaliação funcional de pacientes com a doença de Alzheimer. Brazilian J Phys Ther.

[39] Trentini CM, Yates DB, Heck VS (2014). WASI - Escala Wechsler Abreviada de Inteligência.

[40] Wechsler D (1999). Manual for the Wechsler abbreviated intelligence scale (WASI).

[41] Zimmermann N, Rebouças R, Fonseca RP Questionário de dados socioculturais, médicos e neuropsicológicos para traumatismo cranioencefálico (TCE).

[42] Oldfield RC (1971). The assessment and analysis of handedness: The Edinburgh inventory. Neuropsychologia.

[43] Brito GN, Brito LS, Paumgartten FJ, Lins MF (1989). Lateral preferences in Brazilian adults: an analysis with the Edinburgh Inventory. Cortex.

[44] Associação Brasileira de Empresas de Pesquisa (2008). http://www.abep.org.

[45] Pawlowski J, Remor E, Mattos Pimenta Parente MA, Salles JF, Fonseca RP, Bandeira DR (2012). The influence of reading and writing habits associated with education on the neuropsychological performance of Brazilian adults. Read Writ.

[46] Associação Brasileira de Empresas de Pesquisa (2015). Critério de Classificação Econômica Brasil 2015.

[47] Pachana NA, Byrne GJ, Siddle H, Koloski N, Harley E, Arnold E (2007). Development and validation of the Geriatric Anxiety Inventory. Int Psychogeriatrics.

[48] Massena PN, de Araújo NB, Pachana N, Laks J, de Pádua AC (2015). Validation of the Brazilian Portuguese Version of Geriatric Anxiety Inventory - GAI-BR. Int Psychogeriatrics.

[49] Yesavage J, Brink T, Rose T, Lum O, Huang V, Adey M (1983). Development and Validation of a Geriatric Depression Screening Scale: A preliminary report. J Psychiatr Res.

[50] Almeida OP, Almeida SA (1999). Confiabilidade da versão brasileira da escala de depressão em geriatria (GDS) versão reduzida. Arq Neuropsiquiatr.

[51] Johnson N, Barion A, Rademaker A, Rehkemper G, Weintraub S (2004). The Activities of Daily Living Questionnaire: a validation study in patients with dementia. Alzheimer Dis Assoc Disord.

[52] Holz MR, Kochhann R, Ferreira P, Tarrasconi M, Chaves MLF, Fonseca RP (2017). Cognitive performance in patients with Mild Cognitive Impairment and Alzheimer's disease with white matter hyperintensities: An exploratory analysis. Dement Neuropsychol.

[53] Holz MR, Kochhann R, Cardoso CO, Zimmermann N, Pagliarin KC, Fonseca RP (2018). Frequência de hábitos de leitura e escrita: normas, aplicação, pontuação e interpretação de uma medida sociocultural-linguística da avaliação neuropsicológica. Avaliação de funções executivas e linguagem em adultos.

[54] Casarin FS, Scherer LC, Parente MAPM, Ferré P, Lamelin F, Côté H (2014). Bateria Montreal de Avaliação da Comunicação - versão abreviada - Bateria MAC Breve.

[55] Casarin FS, Scherer LC, Ferré P, Ska B, Parente MAP de M, Joanette Y (2013). Adaptação do Protocole MEC de Poche e da Bateria MAC Expandida: Bateria MAC Breve. Psico.

[56] Dijkstra K, Bourgeois M, Burgio L, Allen R (2002). Effects of a communication intervention on the discourse of nursing home residents with dementia and their nursing assistants. J Med Speech- Lang Pathol.

[57] De Santi S, Koenig L, Obler LK, Goldberger J, Bloom RL, Obler LK, Santi S De, Ehrlich J (1994). Cohesive devices and conversational discourse in Alzheimer's disease. Discourse analysis and applications: Studies in adult clinical populations.

[58] Wright HH, Capilouto GJ, Srinivasan C, Fergadiotis G (2011). Story Processing Ability in Cognitively Healthy Younger and Older Adults. J Speech Lang Hear Res.

[59] Drag LL, Bieliauskas L a (2010). Contemporary Review 2009: Cognitive Aging. J Geriatr Psychiatry Neurol.

[60] Wolf D, Zschutschke L, Scheurich A, Schmitz F, Lieb K, Tüscher O (2014). Age-related increases in stroop interference: Delineation of general slowing based on behavioral and white matter analyses. Hum Brain Mapp.

[61] Verhaeghen P, Cerella J, Basak C (2006). Task Complexity, and Efficiency Modes: The Influence of Working Memory Involvement on Age Differences in Response Times for Verbal and Visuospatial Tasks. Aging, Neuropsychol Cogn.

[62] Davis BH, Guendouzi J, Davis BH, Guendouzi J (2013). Pragmatics in Dementia Discourse.

[63] Dijkstra K, Bourgeois MS, Allen RS, Burgio LD (2004). Conversational coherence: discourse analysis of older adults with and without dementia. J Neurolinguistics.

[64] Duchan J, Bloom RL, Obler LK, DeSanti SD (1994). Approaches to the study of discourse in the social sciences. Discourse analysis and applications.

[65] Schmitter-Edgecombe M (2000). Aging and Word-Finding A Comparison of Spontaneous and Constrained Naming Tests. Arch Clin Neuropsychol.

[66] Marini A, Andreetta S, del Tin S, Carlomagno S (2011). A multi-level approach to the analysis of narrative language in aphasia. Aphasiology.

[67] Glosser G, Deser T (1992). A comparison of changes in macrolinguistic and microlinguistic aspects of discourse production in normal aging. J Gerontol.

[68] Trabasso T, Sperry LL (1985). Causal relatedness and importance of story events. J Mem Lang.

[69] Hendriks P, Koster C, Hoeks JCJ (2014). Referential choice across the lifespan: Why children and elderly adults produce ambiguous pronouns. Lang Cogn Neurosci.

[70] Cohen G (1979). Language comprehension in old age. Cogn Psychol.

[71] Marini A, Boewe A, Caltagirone C, Carlomagno S (2005). Age-related differences in the production of textual descriptions. J Psycholinguist Res.

[72] De Beni R, Palladino P, Borella E, Lo Presti S (2003). Reading comprehension and aging: Does an age-related difference necessarily mean impairment?. Aging Clin Exp Res.

[73] McGinnis D (2009). Text comprehension products and processes in young, young-old, and old-old adults. J Gerontol B Psychol Sci Soc Sci.

[74] Mund I, Bell R, Buchner A (2012). Aging and interference in story recall. Exp Aging Res.

[75] Davis DK, Alea N, Bluck S (2015). The Difference between right and wrong: Accuracy of older and younger adults' story recall. Int J Environ Res Public Health.

[76] Zhuang J, Johnson MA, Madden DJ, Burke DM, Diaz MT (2016). Age-related differences in resolving semantic and phonological competition during receptive language tasks. Neuropsychologia.

[77] Radvansky GA, Dijkstra K (2007). Aging and situation model processing. Psychonomic Bulletin and Review.

[78] Radvansky GA, Zwaan RA, Curiel JM, Copeland DE (2001). Situation models and aging. Psychol Aging.

[79] Anderson S, Parbery-Clark A, White-Schwoch T, Kraus N (2012). Aging Affects Neural Precision of Speech Encoding. J Neurosci.

[80] Helfer KS, Freyman RL (2008). Aging and speech-on-speech masking. Ear Hear.

[81] Pushkar D, Basevitz P, Arbuckle T, Nohara-LeClair M, Lapidus S, Peled M (2000). Social behavior and off-target verbosity in elderly people. Psychol Aging.

[82] Xue SA, Hao GJ (2003). Changes in the human vocal tract due to aging and the acoustic correlates of speech production: a pilot study. J Speech Lang Hear Res.

